# Spring Accumulation Rates in North Atlantic Phytoplankton Communities Linked to Alterations in the Balance Between Division and Loss

**DOI:** 10.3389/fmicb.2021.706137

**Published:** 2021-08-24

**Authors:** Kristina D. A. Mojica, Michael J. Behrenfeld, Megan Clay, Corina P. D. Brussaard

**Affiliations:** ^1^Department of Botany and Plant Pathology, Oregon State University, Corvallis, OR, United States; ^2^Department of Marine Microbiology and Biogeochemistry, Royal Netherlands Institute for Sea Research, Den Burg, Netherlands; ^3^Australian Centre for Ecogenomics, University of Queensland, Brisbane, QLD, Australia

**Keywords:** phytoplankton, bloom dynamics, North Atlantic, Disturbance Recovery Hypothesis, light perturbation

## Abstract

For nearly a century, phytoplankton spring blooms have largely been explained in the context of abiotic factors regulating cellular division rates (e.g., mixed-layer light levels). However, the accumulation of new phytoplankton biomass represents a mismatch between phytoplankton division and mortality rates. The balance between division and loss, therefore, has important implications for marine food webs and biogeochemical cycles. A large fraction of phytoplankton mortality is due to the combination of microzooplankton grazing and viral lysis, however, broad scale simultaneous measurements of these mortality processes are scarce. We applied the modified dilution assay along a West-to-East diagonal transect in the North Atlantic during spring. Our results demonstrate positive accumulation rates with losses dominated by microzooplankton grazing. Considering the dynamic light environment phytoplankton experience in the mixed surface layer, particularly in the spring, we tested the potential for incubation light conditions to affect observed rates. Incubations acted as short-term ‘light’ perturbations experiments, in which deeply mixed communities are exposed to elevated light levels. These “light perturbations” increased phytoplankton division rates and resulted in proportional changes in phytoplankton biomass while having no significant effect on mortality rates. These results provide experimental evidence for the Disturbance-Recovery Hypothesis, supporting the tenet that biomass accumulation rates co-vary with the specific rate of change in division.

## Introduction

Nearly half of the net primary production on Earth is due to phytoplankton in the ocean (Field et al., 1998; Friend et al., 2009). The North Atlantic Ocean is a particular “hot spot” for production, accounting for 20% of the global net ocean CO_2_ uptake (Deser and Blackmon, 1993). Much of this productivity occurs during the recurrent vernal (spring) phytoplankton bloom and accordingly this event has been thoroughly studied for the past century. Traditionally, this bloom has been attributed to elevated springtime phytoplankton division rates caused by mixed-layer shoaling and increased incident sunlight (Gran and Braarud, 1935; Sverdrup, 1953; Follows and Dutkiewicz, 2002; Siegel et al., 2002; Henson et al., 2009). In other words, phytoplankton accumulation rates (*r*), i.e., changes in biomass, are proportional to division rates. However, satellite, *in situ*, and modeling studies have recently revealed that phytoplankton accumulation rates are, in fact, independent of the absolute value of division rate (Behrenfeld, 2010; Behrenfeld and Boss, 2014, 2018). Accumulations in biomass reflect the net balance between the specific rates of phytoplankton division (μ) and loss (*l*) (i.e., *r* = μ – *l*), thus *r* can be independent of μ if μ and *l* covary (Behrenfeld and Boss, 2014, 2018).

Phytoplankton mortality has traditionally been attributed to grazing by zooplankton and the loss of cells from the euphotic zone due to sinking. However, viruses and microzooplankton grazers can also be important sources of mortality (Sherr and Sherr, 2002; Brussaard, 2004; Baudoux et al., 2006). Short micrograzer generation times and viral replication cycles, combined with high rates of micrograzer predation and viral infection, allow these predators to rapidly respond to increases in prey/host abundance. Accordingly, microzooplankton and viruses have the capacity to rapidly collapse a bloom following its climax (Matsuyama et al., 1999; Schroeder et al., 2003; Nagasaki et al., 2004), or even prevent a bloom from happening (Gallegos et al., 1996; Brussaard, 2004; Brussaard et al., 2005). The rapid response time of phytoplankton mortality factors also promotes a tight temporal coupling between phytoplankton division and loss rates, such that daily phytoplankton production in the Northeastern Atlantic during summer is closely matched by collective daily losses of grazing and vial lysis (Caceres et al., 2013; Mojica et al., 2016). Temporal perturbations in growth conditions can cause disturbances in the phytoplankton division-loss balance and be largely responsible for changes in phytoplankton concentrations (Behrenfeld, 2014; Behrenfeld et al., 2017; Behrenfeld and Boss, 2018). Accordingly, in the spring, once the mixed layer stops deepening, phytoplankton and their mortality factors rise in concentration in a parallel fashion. Light-driven increases in division rate and slight lags in the response of predators to these changes in division maintain a growth-loss imbalance allowing for positive accumulation rates that culminate with the annual phytoplankton biomass maximum around May–June (Behrenfeld and Boss, 2014, 2018). However, broad scale simultaneous measurements of microzooplankton grazing and viral lysis during the spring are scarce, particularly in the North Atlantic Ocean, limiting our ability to understand these nuances of phytoplankton bloom dynamics. Moreover, the capability of microzooplankton and viruses to respond to changes in phytoplankton biomass on similar timescales remains unknown. There is evidence, however, that the partitioning of phytoplankton mortality amongst these two modes may be related to mixing processes (Mojica et al., 2016). The impact on marine food dynamics and elemental cycling varies substantially between mortality types (Suttle, 2007; Brussaard et al., 2008; Calbet and Alcaraz, 2009). Therefore, the partitioning of photosynthetic biomass during the accumulation phase has important implications for ecosystem functioning over the entire annual cycle.

During the spring of 2018, we conducted modified dilution experiments along a West-to-East diagonal transect across the North Atlantic providing simultaneous rates of growth and loss of phytoplankton over a range of oceanic provinces and conditions. This allowed us to evaluate whether the balance between division and loss during the spring tends toward positive values for accumulation rates (*r*) within phytoplankton populations. Moreover, concurrent measurements of viral- and grazing-mediated mortality of phytoplankton populations provided information on how phytoplankton mortality was partitioned between these two mortality pathways. Finally, we examine the implications of altering the light environment experienced by phytoplankton by removing phytoplankton from a deeply mixed surface layer and incubating them under static simulated *in situ* conditions.

## Materials and Methods

### Sampling and Physicochemical Variables

**FIGURE 1:**
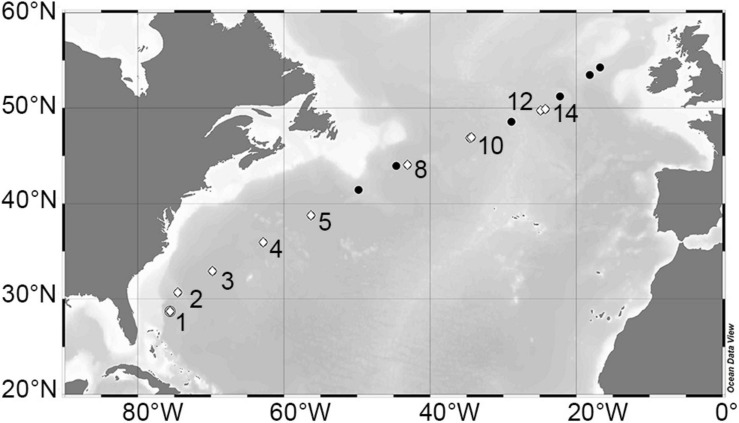
Leg 8 NICO cruise transect in the North Atlantic Ocean. Bathymetric map depicting stations sampled during April of 2018. Modified dilution assays to simultaneously determine viral lysis and microzooplankton grazing rates of phytoplankton were performed at stations (numbered) indicated by white diamond symbols. Figure was prepared using Ocean Data View version 4 (Schlitzer, 2002).

In April (6–28th) of 2018, 17 stations were sampled in the North Atlantic during Leg 8 of the NICO (Netherlands’ Initiative for Changing Oceans) expedition on the R/V Pelagia. The stations traversed diagonally across the North Atlantic Ocean from ∼29°N just off the coast of New Providence (Bahamas) to 54°N off the coast of Galway, Ireland (Figure 1). Water samples were collected at each station using a 24-bottle rosette sampler equipped with 12 L GO-Flow (General Oceanics, Miami, FL, United States) bottles, a standard conductivity, temperature, and depth (CTD) sensor package (Sea-Bird Electronics, Bellevue, WA, United States), and an auxiliary sensor for chlorophyll-a (Chl *a*) autofluorescence (Chelsea Aqua 3 sensor, Chelsea Instruments, West Molesey, United Kingdom). Downcast CTD data were processed using SeaSave software and interpolated to a uniform vertical resolution of 1 m. Sigma-theta (σ_θ_; kg m^–3^), potential temperature (θ;°C), and Brunt-Väisälä frequency (N^2^, s^–2^) were computed using MATLAB in conjunction with the TEOS-10 Gibbs SeaWater (GSW) Oceanographic toolbox (v3.3) (Morgan, 1994; McDougall and Barker, 2011). Sigma-theta is defined as σ_θ_ = ρ_(*S*,θ,0)_−1000, where ρ_(*S*,θ,0)_ is the density of seawater calculated with *in situ* salinity, potential temperature, and a reference pressure of zero. Mixed layer depth (MLD) was defined based on the dynamic threshold method according to Mojica and Gaube (in revision). Specifically, MLD was defined as the depth at which the change in potential density was greater than the standard deviation (σ) of potential density for a vertical profile, given that σ is less than 0.01 kg m^–3^. This method yielded related (current study; *r* = 0.96) but shallower (current study; bias = –11.8 m) estimates compared to the traditional fixed threshold of 0.03 kg m^–3^ (Brainerd and Gregg, 1995; de Boyer Montégut et al., 2004). Moreover, the dynamic threshold method provided more robust estimates of MLD with an average quality index (Lorbacher et al., 2006) of 0.73 compared to 0.55 using the fixed threshold. Water column stratification conditions at each station were classified as “non-stratified” when the average *N*^2^ value for the upper 100 m ($\bar{N^{2}}$) was <2 × 10^–5^ s^–2^, as “weakly stratified” when 2 × 10^–5^ < ($\bar{N^{2}}$) < 5 × 10^–5^ s^–2^, and as “strongly stratified” when ($\bar{N^{2}}$) > 5 × 10^–5^ s^–2^ (Mojica et al., 2015).

Discrete water samples for dissolved inorganic phosphate (PO_4_), ammonium (NH_4_), nitrate (NO_3_), and silicate were gently filtered through 0.2 μm pore size polysulfone Acrodisk filters (32 mm, Pall Inc.), after which samples were stored at –20°C until analysis. Dissolved inorganic nutrients were analyzed onboard using a Bran + Luebbe QuAAtro AutoAnalyzer for dissolved orthophosphate (Murphy and Riley, 1962), inorganic nitrogen (nitrate + nitrite: NO_x_) (Grasshoff, 1983), ammonium (Koroleff, 1969; Helder and de Vries, 1979), and silicate (Strickland and Parsons, 1968).

### Phytoplankton Abundance

A Becton–Dickinson (Erembodegem, Belgium) FACSCalibur flow cytometer (FCM) equipped with an air-cooled 488 nm (15 mW) excitation wavelength Argon laser was used on fresh samples to enumerate photoautotrophic prokaryotic cyanobacteria and eukaryotic phytoplankton (<20 μm cell diameter; size cutoff based on sample injection port diameter). Samples were measured for 10 min using a high flow rate with the discriminator set on red chlorophyll autofluorescence. Phytoplankton populations were distinguished using bivariate scatter plots of autofluorescent properties (orange autofluorescence from phycoerythrin for the cyanobacteria *Synechococcus* spp. and certain cryptophytes and red autofluorescence from Chl *a* for photoautotrophs) against side scatter. The list mode files obtained from FCM were analyzed using the software package FCS Express (v6).

Size-fractionation was performed regularly to provide average cell size for the different phytoplankton subpopulations. Specifically, a whole water sample (15 mL) was size-fractionated by sequential filtration through 12, 10, 8, 5, 3, 2, 1, 0.8, and 0.6 μm pore size polycarbonate filters mounted in a manifold filtration system (Millipore, MA, United States). Each fraction was then analyzed using FCM as described above. The equivalent spherical diameter for each population was determined as the size displayed by the median (50%) number of cells retained for that cluster.

In total, seven different phytoplankton populations were considered in the analysis based on statistically significant abundances within dilution experiment incubation bottles. Phytoplankton populations included two cyanobacterial populations, i.e., *Synechococcus* spp. (average size range of 0.8 ± 0.2 μm) and *Prochlorococcus* (0.7 ± 0.0 μm) and five eukaryotic populations. The photosynthetic eukaryotic populations, differentiated based on variations in side scatter, included two pico-sized groups (≤3 μm): Pico I (0.8 ± 0.2 μm) and Pico II (1.2 ± 0.4 μm) and three nano-sized groups (>3 μm): Nano I (3.3 ± 0.9 μm), Nano II (5.1 ± 0.8 μm), and one group identified as Cryptophycea based on size and presence of phycoerythrin (Crypto; 4.0 ± 0.8 μm). Pico I was distinguished from *Prochlorococus* by a shift in the fluorescence signature (i.e., based on the bivariate plot of red fluorescence versus side scatter) and by its slightly larger size range (Biller et al., 2014).

### Modified Dilution Experiments

At 9 stations along the transect (white diamonds in Figure 1), modified dilution experiments (Kimmance and Brussaard, 2010; Mojica et al., 2016) were conducted onboard to provide simultaneous estimates of viral lysis and microzooplankton (<200 μm) grazing rates for the different photoautotrophic groups. Experiments were conducted using water samples obtained from depths where Chl *a* autofluorscence was maximal [that is, either at the deep chlorophyll maximum (DCM) or within the mixed layer (ML)]. Accordingly, at the two southernmost stations, phytoplankton were sampled from the deep chlorophyll maximum (as determined from subsurface maxima of CTD Chl *a* autofluorescence) and at the seven remaining stations, phytoplankton were sampled from within the mixed layer at ∼15 m. For each experiment, natural seawater was gently passed through a 200-μm mesh to remove mesozooplankton (while retaining microzooplankton) and combined with 0.45 μm diluent or 30 kDa ultrafiltrate in proportions of 100, 70, 40, and 20% to create gradients in microzooplankton and viral-induced mortality, respectively. The 0.45 μm filtrate (microzooplankton grazers removed) was achieved by gravity filtration of natural seawater through a 0.45-μm Sartopore capsule filter with a 0.8-μm prefilter (Sartopore 2300, Sartorius Stedim Biotech, Göttingen, Germany). The 30-kDa ultrafiltrate (microzooplankton grazers and viruses removed) was generated by tangential flow filtration using a polyethersulfone membrane (Vivaflow 200, Sartorius Stedim Biotech, Göttingen, Germany). After preparation of the two parallel dilution series (12 bottles each), a 3-ml subsample was taken and phytoplankton were enumerated by FCM (see above). The sample volume was replaced with water of identical dilution and closed with a convex inlayed cap to prevent the introduction of air bubbles that cause turbulence and reduce grazing activity. The 1-L polycarbonate bottles were then mounted onto a slow turning incubation wheel (manufactured for C.P.D.B. by the National Marine Facilities at NIOZ) in an on-deck flow-through seawater incubator and incubated for 24 h at *in situ* temperature and at a light level approximating the *in situ* light intensity at the depth of sampling (incubation light levels were created using neutral density screen). After the 24 h incubation period, a second FCM phytoplankton count was conducted and the resulting apparent growth rate for each phytoplankton group was determined.

Microzooplankton grazing rate was estimated from the regression coefficient of the apparent growth rate versus fraction of natural seawater for the 0.45 μm series. Similarly, total mortality rate (i.e., combined rate of viral-induced lysis and microzooplankton grazing) was determined from a regression of the 30 kDa series (Baudoux et al., 2006; Kimmance and Brussaard, 2010). A significant difference between the two regression coefficients for each series (assessed by analysis of covariance, i.e., ANCOVA) indicates a significant viral lysis rate. Phytoplankton gross division rate (μ, no mortality) was derived from the y intercept of the 30 kDa series regression.

The viral lysis and grazing rates were analyzed with a two-way analysis of variances (ANOVA) with type III sum of squares to assess differences between the two sources of mortality (i.e., viral lysis versus grazing) and among the different phytoplankton groups (i.e., cyanobacteria, picoeukaryotes, and nanoeukaryotes). Model assumptions were confirmed using the Brown–Forsythe test for homogeneity of variance and Shapiro–Wilk test for normality.

Mortality rates were (y)^–2^ transformed to fit model assumptions. Statistical analysis was implemented in R (R Development Core Team, 2012) using the “car” package (Fox et al., 2016) with a significance level (α) of 0.05. Total mortality and division rates were also analyzed with a two-way ANOVA with type III sum of squares to assess differences between rates (and therefore significance of accumulation rates) and among the different phytoplankton groups. Rates were square root transformed to fit model assumptions. *Post hoc* comparisons of significance were evaluated based on Tukey’s honest significant differences test.

### Light Conditions

Daily photosynthetically available radiation (PAR; mole photons m^–2^ day^–1^) from the Moderate Resolution Imaging Spectroradiometer (MODIS) was downloaded from the OceanColor Web^1^ and extracted at a resolution of ^1^/_12_ degree latitude × ^1^/_12_ degree longitude for each station location. Diffuse attenuation for PAR [K_*d*_(PAR)] was calculated from shipboard fluorescence-based Chl *a* measurements following Morel et al. (2007). *In situ* growth irradiance was defined as PAR at the depth of sampling (PAR_z_) for samples from the DCM and as the median mixed layer light level (PAR_mld_) for samples within the ML (Behrenfeld et al., 2005). PAR_z_ and PAR_mld_ were then calculated as:

$$PAR\left(\mathrm{mole}\mathrm{photons}m^{-2}h^{-1}\right)=\left[\left({\bar{PAR}}_{1}*0.975\right)e^{-\text{kd}\left(\text{PAR}\right)*\text{z}}\right]/dayL$$

where 0.975 is the surface reflectance correction (Austin, 1974), ${\bar{PAR}}_{1}$ is the average daily PAR for a given location over the 3 days prior to station arrival, day length (dayL) is the number of hours of daylight at the station location, and depth (z) is either the DCM sampling depth or MLD/2 for samples originated from the ML. Here, we used the 3-day average PAR because we are interested in division rates for populations acclimated to their variable *in situ* light conditions.

Incubation light levels were calculated as:

$$PAR\left(\mathrm{mole}\mathrm{photons}m^{-2}h^{-1}\right)=\frac{\left(\bar{PAR_{2}}*0.975\right)}{dayL}*\%Irr$$

where $\bar{PAR_{2}}$ is the daily average PAR at the station location during incubation and %*Irr* is the percentage of PAR transmitted through the neutral density screening applied to a given incubation bottle (i.e., measured in incubator after neutral density screening) relative to PAR just below the surface (i.e., measured *in situ*). Light levels for incubations (i.e., %*Irr*) were based on ambient light conditions at the depth of sampling (i.e., DCM depth for DCM samples and 15 m for ML samples) (Table 1). Light measurements for %*Irr* were determined from onboard measurements obtained using LI-COR LI 193SA Underwater Spherical Quantum Sensor. In order to get sufficient satellite PAR data for each station (which are not always available), $\bar{PAR_{2}}$ for incubations was calculated using PAR values averaged over 48 h. Delta PAR (ΔPAR) was calculated as the difference between PAR for the incubations and the *in situ* PAR (PAR_z_ or PAR_mld_).

**TABLE 1:** Station location, day of year (DOY), sampling depth, and *in situ* biological and physiochemical characteristics, including mixed layer depth (MLD), 100 m depth averaged Brunt–Väisälä frequency (N^2^), and stratification of the water column during the spring NICO leg 8 cruise.

**Station No.**	**DOY**	**Latitude (°N)**	**Longitude (°E)**	**Day length (h)**	**MLD (m)**	**N^2^ (s^–2^)**	**Stratification**	**Z_eu_ (m)**	**Sample depth (m)**	**% Irr**	**Temperature (°C)**	**Salinity**	**Chl *a* autofluorescence (μg L^–1^)**	**Total Phytoplankton (×10^4^ mL^–1^)**	**Nutrients (μM)**			
															**PO_4_**	**NO_3_**	**NH_4_**	**Silicate**
1	97	28.7	−75.6	12.2	19	2.5 × 10^–5^	Weakly stratified	102	78	0.9	21.9	36.8	0.24	9.58	0.05	0.53	0.29	0.76
2	98	30.7	−74.0	12.2	32	2.9 × 10^–5^	Weakly stratified	93	75	4.5	22.2	36.8	0.29	7.34	0.02	0.40	0.09	0.72
3	100	32.9	−69.8	12.3	23	1.0 × 10^–5^	Non-stratified	58	15	25.0	19.7	36.7	0.24	4.78	0.04	0.09	0.11	0.71
4	102	35.9	−62.8	12.4	76	2.3 × 10^–6^	Non-stratified	64	15	28.6	19.6	36.7	0.21	2.91	0.09	0.96	0.44	0.80
5	104	38.8	−56.3	12.5	245	−2.0 × 10^–7^	Non-stratified	56	15	20.0	19.0	36.6	0.29	1.96	0.07	5.38	0.17	0.95
8	108	44.0	−43.1	12.9	50	1.7 × 10^–5^	Non-stratified	37	15	20.0	9.9	34.6	0.85	1.59	0.33	2.66	0.28	2.25
10	110	46.9	−34.3	13.1	205	−3.5 × 10^–8^	Non-stratified	52	15	15.0	12.5	35.7	0.34	3.00	0.52	8.33	0.12	3.50
12	112	49.7	−24.9	13.3	69	2.6 × 10^–6^	Non-stratified	37	15	20.0	10.6	35.3	0.78	4.07	0.65	10.76	0.08	4.37
14	113	49.9	−24.2	13.4	78	2.4 × 10^–6^	Non-stratified	39	15	15.0	11.1	35.4	0.72	4.86	0.62	10.61	0.09	4.11

*Euphotic zone depth (Z_eu_) defined as the depth at which light is 1% of its surface value and calculated according to Morel et al. (2007). Light levels for incubations (i.e.,%Irr) is the percentage of PAR transmitted through the neutral density screening applied to a given incubation bottle (measured in incubator after neutral density screening) relative to PAR just below the surface (measured *in situ*).%Irr was targeted to match ambient light conditions at sample depth.*

## Results

### Environmental Variability

**FIGURE 2:**
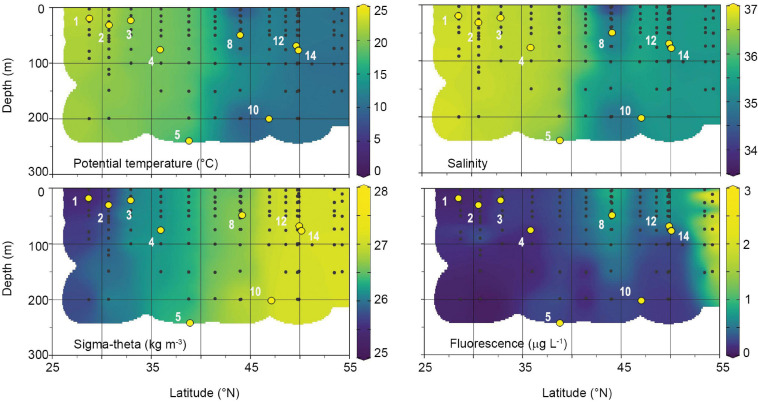
Physical and chlorophyll fluorescence characteristics of the water column during the April 2018 Leg 8 NICO expedition. Black dots represent individual sampling points. White numbers are station numbers where modified dilutions experiments were performed. Yellow points indicate MLD of stations.

In April 2018, the water column along the Leg 8 NICO cruise track was characterized as weakly stratified to well-mixed (Table 1). Physicochemical parameters were relatively uniform with depth at a given station in the upper 200 m, but varied significantly with latitude (Figure 2 and Table 1). In the southern half of the transect (28–36°N), potential temperature (θ), salinity, and sigma-theta (σ_θ_) averaged 20.7 ± 1.3°C, 36.7 ± 0.1, and 25.9 ± 0.3 kg m^–3^, respectively. Dissolved inorganic nutrient concentrations in the surface were low and uniform in this region, with averages of 0.50 ± 0.40 μM for nitrate, 0.04 ± 0.02 μM for phosphate, 0.70 ± 0.10 μM for silicate. North of 38°N, nutrients increased gradually to reach maximal concentrations of 10.3 μM for nitrate, 0.60 μM for phosphate, and 4.30 μM for silicate. The region between 36 and 44°N exhibited the largest horizontal gradients in physical parameters (Figure 2), with average θ, salinity, and σ_θ_ of 17.5 ± 1.7°C, 36.3 ± 0.4, and 26.4 ± 0.1 kg m^–3^, respectively. In the north (44–54°N), θ and salinity were minimal and σ_θ_ maximal, with averages of 11.1 ± 1.4°C, 35.3 ± 0.4, and 27.0 ± 0.2 kg m^–3^, respectively. MLDs were highly variable, ranging from 19 to 245 m (Table 1). The first two stations were classified as weakly stratified based on 100 m depth averaged Brunt–Väisälä frequencies ($\bar{N^{2}}$) of 2.5 and 2.9 × 10^–5^ s^–2^, respectively. Accordingly, these stations exhibited relatively shallow mixed layer depths of 19 and 32 m and an average subsurface fluorescence maximum of 0.27 centered around 75 m (Figure 2). The remaining stations were classified as non-stratified. Stations 5 and 10 were the most unstable, with low and negative ($\bar{N^{2}}$) and MLD greater than 200 m. Fluorescence was uniformly distributed over the mixed layer at both stations, averaging ∼0.30 ± 0.01 μg L^–1^. Of the non-stratified stations, 3 and 8 had the highest ($\bar{N^{2}}$) and shallowest MLD of 23 and 50 m. Fluorescence in the mixed layer averaged 0.23 ± 0.01 and 0.87 ± 0.03 μg L^–1^, respectively. Station 4 had an intermediate MLD of 76 m and a fluorescence of 0.21 μg L^–1^. Stations 12 and 14 had MLDs of 69 and 78 m, with relatively high fluorescence values of 0.78 and 0.72 μg L^–1^, respectively.

### Phytoplankton Abundance and Composition

Highest phytoplankton concentrations were measured in the DCM of weakly stratified stations (9.6 and 7.3 × 10^4^ cells mL^–1^ for stations 1 and 2, respectively). The phytoplankton populations in the DCM were dominated by cyanobacteria species with *Prochlorococcus* comprising 80 and 51% and *Synechococcus* 19 and 36% of the total counts of Station 1 and 2, respectively. At non-stratified stations, the ML was sampled. At station 4, the ML was comprised mainly of *Prochlorococcus* (46%) and *Synechococcus* (44%). The deep mixed layer of station 5 was predominately comprised of Pico II at 49% of the total counts, followed by the cyanobacteria at 41% and Nano I at 7%. North of 44°N, *Prochlorococcus* was no longer present and Pico I emerged. However, *Synechococcus* continued to comprise a large portion of the phytoplankton community in the ML for the remaining stations. At stations 8 and 10, *Synechococcus* comprised 61 and 69% of the total counts, followed by Pico I at 29 and 26%, respectively. The prevalence of *Synechococcus* decreased to 49% at station 12 and 14, trailed closely by Pico I at 45%. Nano II and Cryptophytes comprised ∼1% or less of the total counts at all stations and depths measured. In general, the total phytoplankton abundance and the relative abundance of the different phytoplankton groups were consistent between communities in incubations and *in situ* (≥96%). One notable exception was station 12, where the incubation community was enriched with *Synechococcus* (i.e., 93% compared to 49% *in situ*) and completely depleted of Pico I (i.e., compared to 45% *in situ*). The water samples used for *in situ* phytoplankton community analysis and for the dilution experiments did not originate from the same CTD bottle, which may have caused this discrepancy.

### Growth Versus Loss of Phytoplankton

Phytoplankton gross division rate (μ) and the contribution of viral lysis and grazing to the mortality of the different phytoplankton groups were assessed at 9 stations of the Leg 8 NICO cruise using the modified dilution method. Results were evaluated for evidence of negative effects of the experimental manipulations on phytoplankton performance (e.g., changes in community composition, dilution induced nutrient limitation, dilution induced losses in growth, etc.). Based on this analysis, the experiment at station 3 was deemed unsuccessful across all phytoplankton groups, as all groups exhibited lower growth rates with increased dilution. In addition, we found reductions in growth in the 20% fraction at 3 stations north of 35°N (i.e., 4, 5, and 15). Interestingly, reductions were consistent across all phytoplankton groups and not restricted to the 30 kDa series where potential enhanced nutrient limitation would be greatest due to reduced remineralization (i.e., due to removal of bacteria and grazers), nor were they restricted to nutrient limited regions of the transect. At these stations, the 20% fraction was excluded from analysis.

**FIGURE 3:**
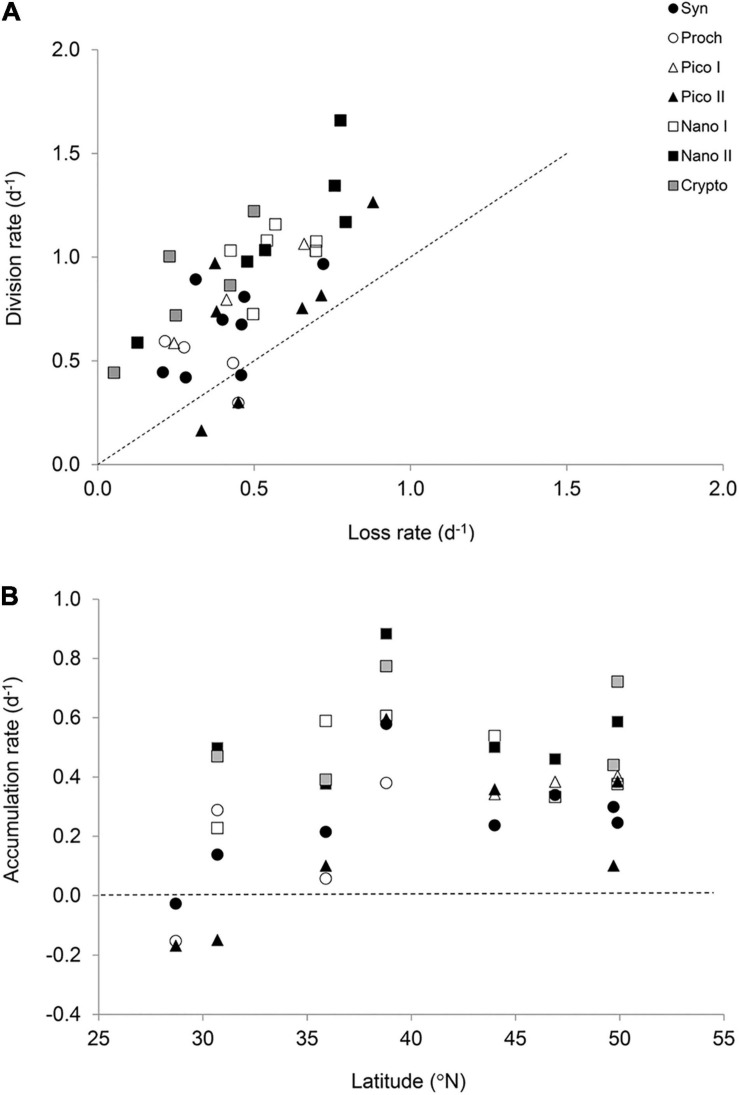
The balance between division and loss rates of the seven phytoplankton groups (<20 μm). **(A)** Relationship between the total loss rate (grazing + viral lysis) and gross growth rate of phytoplankton obtained from the modified dilution method. The dotted line indicates a 1:1 relationship. **(B)** Accumulation rates (i.e., μ – *l*) of phytoplankton across the latitudinal transect. The dotted line indicates no net accumulation.

**FIGURE 4:**
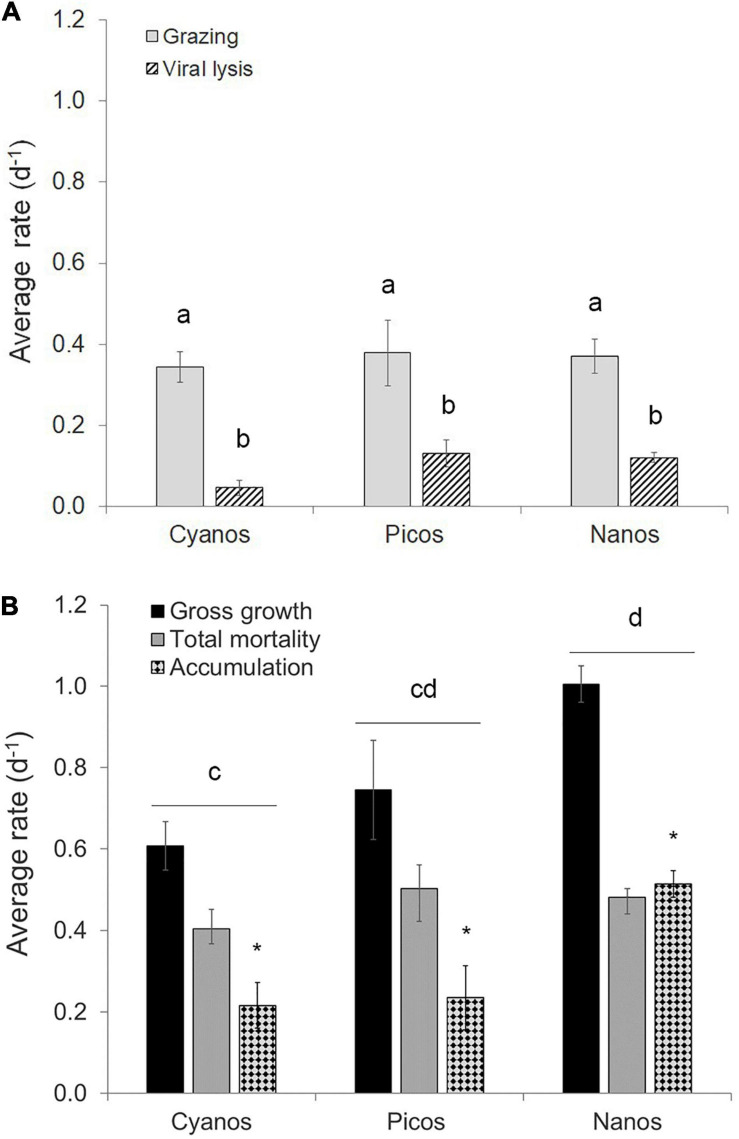
Average rates of division and loss obtained from the modified dilution method for cyanobacteria, and pico- and nano-eukaryotic phytoplankton groups. **(A)** Grazing and viral lysis rates. **(B)** Gross division, total mortality (i.e., grazing and viral lysis), and accumulation rates of the different phytoplankton groups. The different phytoplankton groups include cyanobacteria (cyanos, sample size *N* = 12), picoeukaryotes (picos, *N* = 10), and Nano I (*N* = 17). Error bars represent standard error. In each panel, bars with different letters are significantly different (*p* < 0.05), as tested by two-way analysis of variance. In panel B, line and associated letter represent the results of *post hoc* comparison of the means using Tukey’s honest significant difference. The asterisks represent the significance of the positive accumulation rates as revealed by two-way ANOVA.

Phytoplankton gross growth (or division) rates ranged from 0.16 to 1.66 day^–1^ and were in excess of total mortality (i.e., viral lysis + grazing) which ranged from 0.05 to 0.88 day^–1^ (Figure 3A). This resulted in predominately positive accumulation rates with a median value of 0.38 day^–1^ and range of –0.17 to 0.88 day^–1^ (Figure 3B). Phytoplankton mortality was generally dominated by microzooplankton grazing, which comprised on average 77 ± 25% of the total phytoplankton mortality. Individual grazing rates varied from 0.01 to 0.65 day^–1^ (Supplementary Figure 1A) with a median value of 0.36 day^–1^. In addition, there was very little variation in the average rates between the different phytoplankton size classes (Figure 4A). Indeed, two-way analysis of variance of the mortality rates revealed a significant main effect of mortality source (*F* = 34.4, *p* < 0.001), whereas the main effect of phytoplankton group (*F* = 0.0, *p* > 0.05) and the interaction term (*F* = 1.6, *p* > 0.05) were both non-significant. In other words, individual mortality rates were comparable across the different phytoplankton groups (Figure 3A). Viral lysis rates, which varied from 0.0 to 0.48 day^–1^ (Supplementary Figure 1B), were significantly lower than microzooplankton grazing rates with a median value of 0.09 day^–1^. The two-way ANOVA of total mortality and division rates revealed a significant main effect of rate type (*F* = 5.5, *p* = 0.02) and phytoplankton group (*F* = 7.4, *p* = 0.001) with no significant interaction (*F* = 2.7, *p* > 0.05). That is, division rates were significantly greater than total mortality rates, indicating significant positive accumulation rates (Figure 4B). Additionally, rates of division and total mortality of nano-sized eukaryotes were significantly higher than cyanobacteria, with average rates of 1.01 ± 0.05 and 0.48 ± 0.02 day^–1^ compared to 0.61 ± 0.06 and 0.41 ± 0.05, respectively.

### Light and Phytoplankton Growth

**FIGURE 5:**
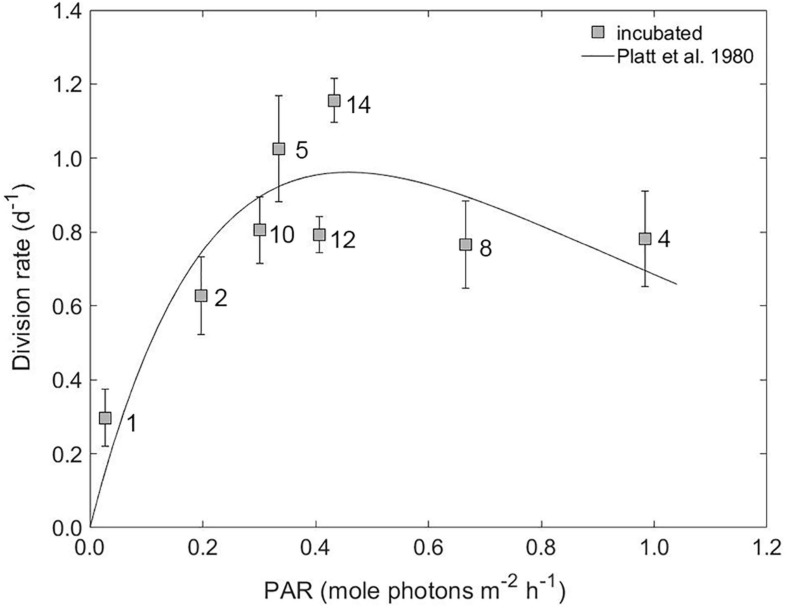
The average gross division rate of phytoplankton groups versus the light level during experimental incubations. The solid line is a fit of the photosynthesis-irradiance (P-I) model of Platt et al. (1980) to our division rate versus PAR data. Station numbers are indicated on plot. Error bars represent standard error.

**FIGURE 6:**
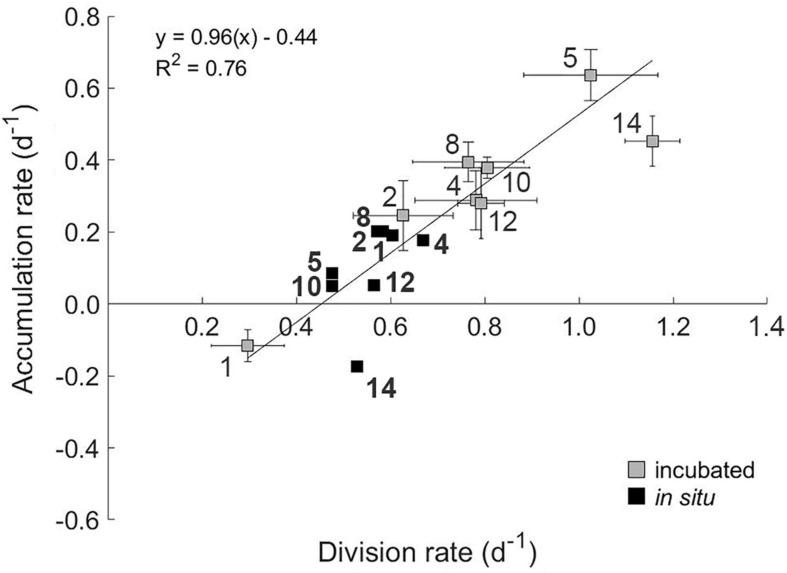
The relationship between accumulation rate and gross division rate of phytoplankton communities subjected to a light disturbance during experimental incubations (gray squares). Station number are indicated on plot. Error bars represent standard error. A geometric mean regression of division rates within the phytoplankton community during dilution incubations and PAR levels <0.5 mole photons m^–2^ h^–1^ was used to calculate the growth of the phytoplankton at *in situ* light levels (black squares). Station numbers associated with *in situ* division rates are shown in bold.

At all stations, aside from Station 1, irradiance levels in the modified dilution incubations were higher than calculated *in situ* growth irradiance (Supplementary Figure 2). Thus, ΔPAR values (i.e., PAR of incubation - PAR *in situ*) were predominately positive, ranging from 0.14 to 0.89 mole photons m^–2^ h^–1^. The exception was Station 1, which had a ΔPAR of –0.04 mole photons m^–2^ h^–1^. The average division rates of the phytoplankton community increased in proportion to PAR at values less than 0.5 mole photons m^–2^ h^–1^ (Figure 5). Station 12, however, had a lower μ than would be expected from this relationship, presumably due to alternations in the composition of the incubation community (see section “Phytoplankton Abundance and Composition”). At larger PAR values, such as those measured at Station 4 and Station 8, associated μ values averaged 0.8 day^–1^. The trend in division rate with PAR is reminiscent of a typical photosynthesis-irradiance (P-I) curve used to describe photosynthesis by phytoplankton as a function of light. Such curves are characterized by an initial light-limited linear response that subsequently saturates at higher light and then often exhibits a downturn from photoinhibition at very high light. For our relationship between μ and incubation light level (Figure 5), a type II geometric mean regression model (Legendre and Legendre, 1998) was applied to define the predicative functional relationship between the division rate and PAR for data less than 0.5 mole photons m^–2^ h^–1^ (Supplementary Figure 3). This relationship was then applied to estimate gross division rates under *in situ* light conditions. At all stations, except Station 1, the resulting *in situ* μ values were lower than incubation μ (Figure 6). At Station 1, *in situ* μ was 2-fold higher than incubation μ.

**FIGURE 7:**
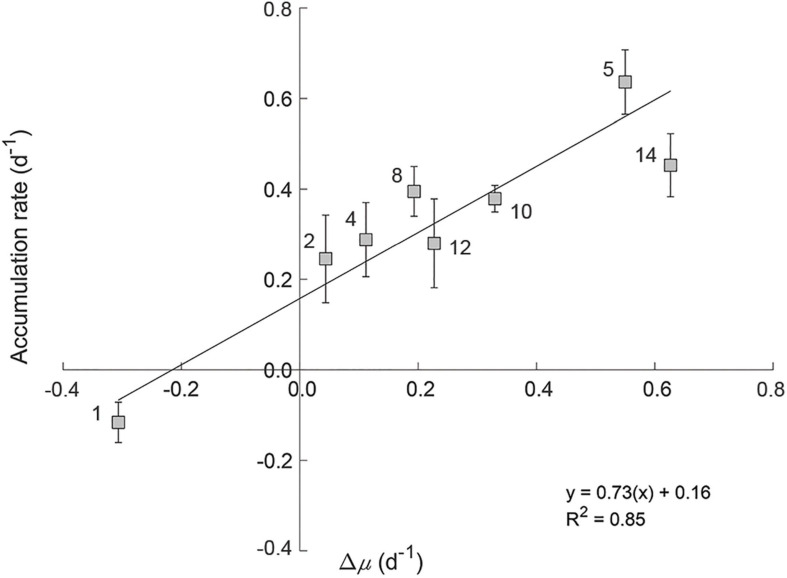
The relationship between the rate of change in division rate (Δμ) and measured accumulation rates of the phytoplankton communities within experimental incubations. Station numbers are indicated on plot. Error bars represent standard error.

We observed no significant effect of PAR on measured loss rates (*l*) (grazing; *p* = 0.22, *R*^2^ = 0.04 and viral lysis rate; *p* = 0.39, *R*^2^ = 0.02). Accordingly, incubation *l* values were used to calculate *in situ* accumulation rates (*r*). No significant relationship was found between *in situ* values for *r* and μ (*R*^2^ = 0.25, *p* = 0.20, black symbols in Figure 6), indicating a coupling between phytoplankton division and loss rates *in situ*. However, a strong linear relationship (*R*^2^ = 0.76) is observed between *r* and μ for the incubation light levels (Figure 6). As incubation light levels were almost always greater than calculated *in situ* (Supplementary Figure 2) and *l* was unaffected by these light changes, this finding suggests that associated accelerations in division rate defined resultant accumulation rates. For stations 2 through 14, these accelerations [i.e., positive specific rate of change in growth (Δμ = incubation μ – *in situ* μ)] ranged from 0.04 to 0.63 day^–1^ (Figure 7). Conversely, at Station 1 where incubation PAR was less than *in situ*, Δμ was –0.31 day^–1^. Measured accumulation rates during the incubations were linearly related to Δμ across this range of measured values.

## Discussion

During the spring of 2018, the water column along our diagonal cruise track in the North Atlantic ranged from weakly stratified to non-stratified, resulting in fairly uniform vertical profiles of physicochemical parameters down to around 200 m. This is consistent with observations of high latitude regions of the North Atlantic remaining well-mixed in the upper 200 m between December and April (van Aken, 2000). Mixed layer depths in this study were in the range of the climatological monthly mean values and variability expected for the North Atlantic (Monterey and Levitus, 1997; de Boyer Montégut et al., 2004; Carton et al., 2008). In accordance with the Disturbance Recovery Hypothesis (DRH) (Behrenfeld and Boss, 2018), once the mixed layer stops deepening, phytoplankton growth increases due to elevated irradiance and at the same time losses increase as more phytoplankton cells allow for enhanced contact rates between predators (zooplankton or viruses) and phytoplankton. Positive accumulation rates are then the result of a combination of light driven increases in phytoplankton division and slight temporal lags in the response of mortality agents to these changes in division (Behrenfeld and Boss, 2014, 2018; Smith et al., 2015). Indeed, our results revealed a relatively strong coupling between division and total mortality rates (i.e., viral lysis + microzooplankton grazing), with loss accounting for an average of 60–70% of phytoplankton growth. Moreover, despite the broad geographical range of our measurements, phytoplankton generally possessed positive rates of accumulation (*r*).

### Partitioning Phytoplankton Mortality

Phytoplankton mortality was dominated by microzooplankton grazing. Absolute grazing rates were comparable to Chl *a-*based grazing rates for the North Atlantic, as well as rates measured during the May–June blooming period (Verity et al., 1993; Gifford et al., 1995; Schmoker et al., 2013; Morison et al., 2019). In addition, we found no significant difference in the specific grazing rates of the different phytoplankton populations, suggesting non-selective grazing. Conversely, rates of viral mediated mortality were nearly 2-fold lower than the average rates reported during the summer in the northeastern North Atlantic (Mojica et al., 2016). Moreover, the average viral lysis to grazing rate ratio (V:G) of phytoplankton groups decreased significantly over the latitudinal gradient (*r* = –0.73, *p*-value = 0.02) (Supplementary Figure 1C), consistent with decreased depth-averaged N^2^. This finding corresponds well with results of Mojica et al. (2016), which demonstrated a shift from viral-lysis dominated mortality at low latitude regions to a grazing-dominated mortality at high latitude regions of the North Atlantic during the summer. In that study, the decrease in viral lysis rates was inversely associated with the vertical mixing coefficient (K_T_; temperature eddy diffusivity). Accordingly, increased vertical mixing appears to be proportionally disadvantageous for effective viral control of phytoplankton populations.

The activity of microzooplankton grazers and viruses are ultimately regulated by the rate at which they encounter/contact a phytoplankton cell (Talmy et al., 2019). Thus, when the mixed layer deepens and entrains phytoplankton-free water, it dilutes the mixed layer population and mortality will be negatively affected as a consequence of reduced encounter rates. As obligate parasites, however, viruses are also completely dependent upon their host for all their metabolic needs (i.e., molecular building blocks and energy). Consequently, suboptimal host growth conditions may negatively affect virus infection and progeny production (Mojica and Brussaard, 2014). This is highlighted by the roles in which light can regulate viral infection dynamics in photosynthetic hosts. Light availability can influence viral adsorption, latent period and burst size, with low light prolonging the latent period and reducing burst size (Cseke and Farkas, 1979; Juneau et al., 2003; Brown et al., 2007; Baudoux and Brussaard, 2008; Jia et al., 2010; Maat et al., 2016; Thamatrakoln et al., 2019). Consequently, phytoplankton experiencing light-limited conditions within a deep mixing layer would likely be inadequate hosts for efficient viral production. Viruses typically have a narrow host specificity, with most viruses infecting only one host species, and therefore can be also be affected by factors regulating phytoplankton diversity (Ostfeld and Keesing, 2012; Short, 2012). Disturbance is expected to initially increase diversity by favoring the less competitive faster growing organisms (Behrenfeld et al., 2021). Indeed, a recent study in the North Atlantic reveals phytoplankton diversity, based on amplicon sequence variants of the V1–V2 region of the 16S rRNA gene, was highest during the accumulation phase of the annual cycle (March–April) and declined during the bloom phase (May–June) (Karp-Boss et al., 2020). Accordingly, viruses would have a lower percentage of success arising from contact to a phytoplankton cell relative to their grazing counterparts (i.e., mainly restricted by size limitations) during the accumulation phase. Viral infection is most effective when their host abundance is high and growth conditions are optimal, and host diversity is relatively low. The impact of viruses can therefore be expected to be most noticeable during enhanced phytoplankton density, such as found during the final stages of blooms development. As such, viruses may play a particularly important role in closing the gap between division and loss as phytoplankton transition from the spring accumulation phase to the summer equilibrium phase (i.e., division and losses in a near balanced state) of the annual phytoplankton cycle, driven by the efficiency of infection rising in parallel to the shoaling of the mixed layer.

### Implications of Light Conditions

As anticipated for spring, the balance between phytoplankton division and loss tends toward positive accumulation rates. A notable attribute of the measured accumulation rates is that their projection in time would indicate that full bloom climax concentrations would be reached on the order of days. This timescale implies a major decoupling of phytoplankton division and loss rates and is surprising given that bloom development in the North Atlantic typically takes place over much longer time periods (commonly months) (Behrenfeld et al., 2013). While we acknowledge that this projection does not account for the likelihood that viral induced mortality increases in parallel with light driven-increases in phytoplankton division, our results indicate that increased light availability in incubations relative to *in situ* could largely account for this discrepancy. Phytoplankton within the mixed layer are continually exposed to changes in their light environment as they are moved vertically through the water column by turbulent mixing. Consequently, the more static light conditions of incubations (only reflecting natural light fluctuations by cloud cover) targeting the light level at the depth of sampling, resulted in changes in PAR that were by-in-large positive. The division rate of the phytoplankton communities within the incubations responded to the change in PAR relative to their *in situ* light level in a manner reminiscent of a typical photosynthesis-irradiance (P-I) curve (Figure 5). Indeed, a positive linear relationship between PAR (i.e., incubation PAR) and gross division rate (μ) can be seen for PAR less than 0.5 mole photons m^–2^ h^–1^, suggesting that these phytoplankton populations were light limited *in situ*. At supersaturating PAR, division rate declined to 0.8 day^–1^, most likely as a consequence of photoinhibition.

During the phytoplankton annual cycle, accelerations and decelerations in division arise from “bottom up” factors (i.e., light or nutrient availability) and can act as a form of disturbance influencing the balance between division and loss rate (Behrenfeld and Boss, 2018). In the present study, our incubations essentially emulated the effect of accelerating division rates through ‘light perturbations’ that caused incubation division rates to differ from *in situ* rates. The influence of these perturbations on the balance between division and loss was apparent in the lack of a corresponding response in phytoplankton mortality. In other words, phytoplankton growth was accelerated and decoupled from mortality, resulting in enhanced accumulation rates. These results provide a clear experimental demonstration of a central element of the Disturbance Recovery Hypothesis, specifically that phytoplankton accumulation rates (*r*) are quantitatively linked to the rate of change in division (Δμ) (Behrenfeld and Boss, 2018).

## Data Availability Statement

The raw data supporting the conclusions of this article will be made available by the authors, without undue reservation.

## Author Contributions

CB conceived the NICO Leg 8 cruise plan and original experimental design. CB and MC collected the samples and executed experiments in the field, and performed the initial FCM analysis. KM, MB, and CB devised the concept and design of the manuscript. KM performed the final analysis and drafted the manuscript. All authors contributed the revision and final editing of the manuscript and were aware of, accept responsibility for this manuscript, and have approved the submitted manuscript.

## Conflict of Interest

The authors declare that the research was conducted in the absence of any commercial or financial relationships that could be construed as a potential conflict of interest.

## Publisher’s Note

All claims expressed in this article are solely those of the authors and do not necessarily represent those of their affiliated organizations, or those of the publisher, the editors and the reviewers. Any product that may be evaluated in this article, or claim that may be made by its manufacturer, is not guaranteed or endorsed by the publisher.

## Footnotes

1
http://oceancolor.gsfc.nasa.gov
