# Supplementary material for: Spring Accumulation Rates in North Atlantic Phytoplankton Communities Linked to Alterations in the Balance Between Division and Loss
Source: Front Microbiol. 2021 Aug 24;12:706137. doi: 10.3389/fmicb.2021.706137 (PMC8422905; doi:10.3389/fmicb.2021.706137)
Supplement: Supplementary file 1 [file Data_Sheet_1.PDF]

frontiers

Supplementary Material

Spring accumulation rates in North Atlantic phytoplankton communities linked to alterations in the balance between division and loss

Kristina D.A. Mojica[1,2*], *Michael J. Behrenfeld*[2], *Megan Clay*[3,4], *Corina P.D. Brussaard*[3]

[1] Current affiliation: Division of Marine Science, School of Ocean Science and Engineering, The University of Southern Mississippi, Stennis Space Center, Mississippi, USA

[2] Department of Botany and Plant Pathology, Cordley Hall 2082, Oregon State University, Corvallis, Oregon 97331-29052, USA

[3] Department of Marine Microbiology and Biogeochemistry, Royal Netherlands Institute for Sea Research (NIOZ), P.O. Box 59, 1790 AB Den Burg, Texel, The Netherlands

[4] Australian Centre for Ecogenomics, Level 5, Molecular Biosciences Bldg, University of Queensland, St Lucia QLD 4072, Brisbane, Australia

*** Correspondence:**
Kristina Mojica
Kristina.Mojica@usm.edu

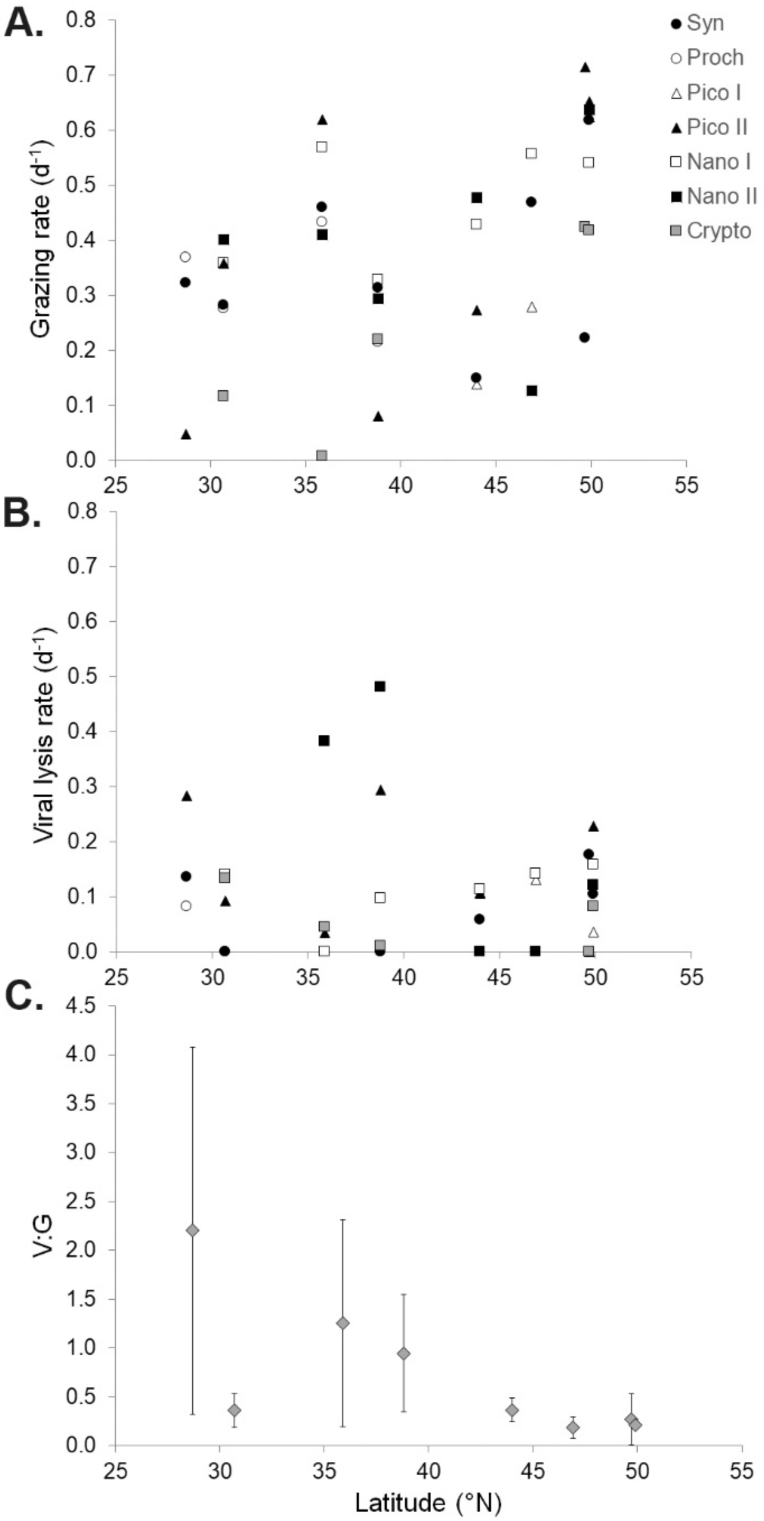

Figure S1. Measured loss rates of seven phytoplankton groups (<20μm). (A) Grazing rate, (B) viral lysis rate, and (C) the average virus to grazing rate ratio of phytoplankton across the latitudinal transect. Error bars represent standard error.

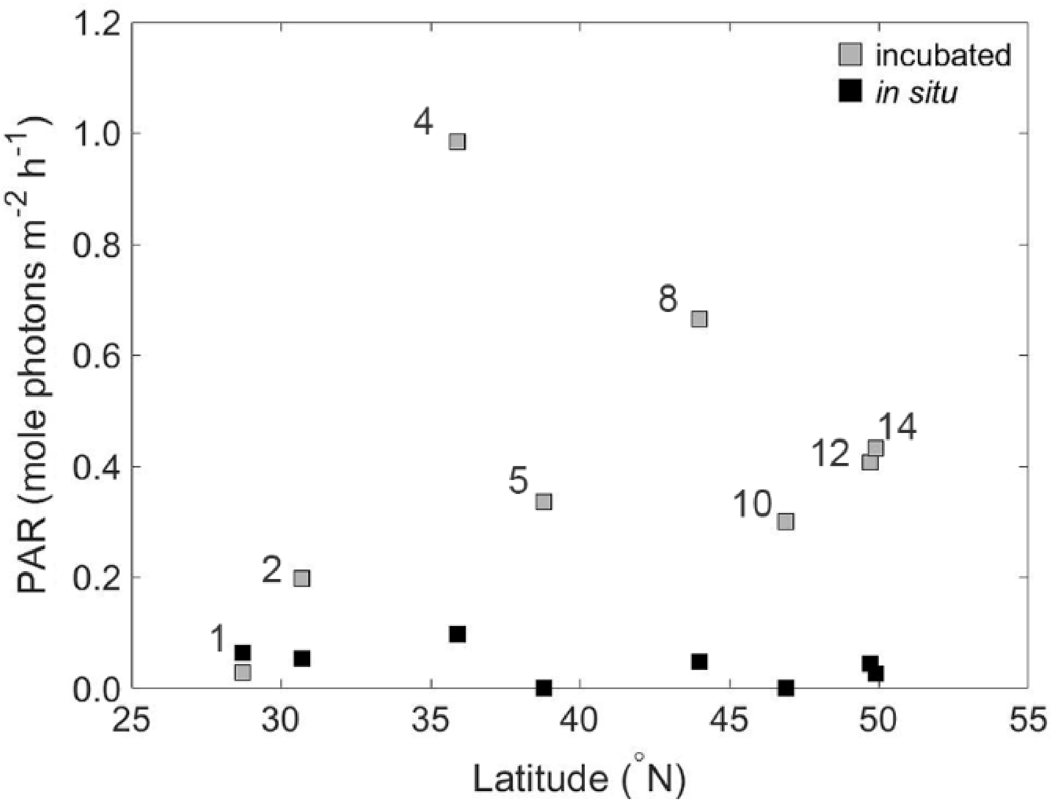

Figure S2. Light conditions of dilution experiment incubations relative to *in situ* (*e.g.,* acclimated) conditions for all experiments conducted during the spring NICO leg 8 cruise. Light levels *in situ* were defined as PAR at the sampling depth (PAR_z) for samples from the DCM and as the median mixed layer light level (PAR_{mld}) for samples within the ML. Incubation light levels were calculated as the product of the average hourly irradiance and the percentage of PAR transmitted through the neutral density screening applied to a given incubation. Station numbers are indicated on plot.

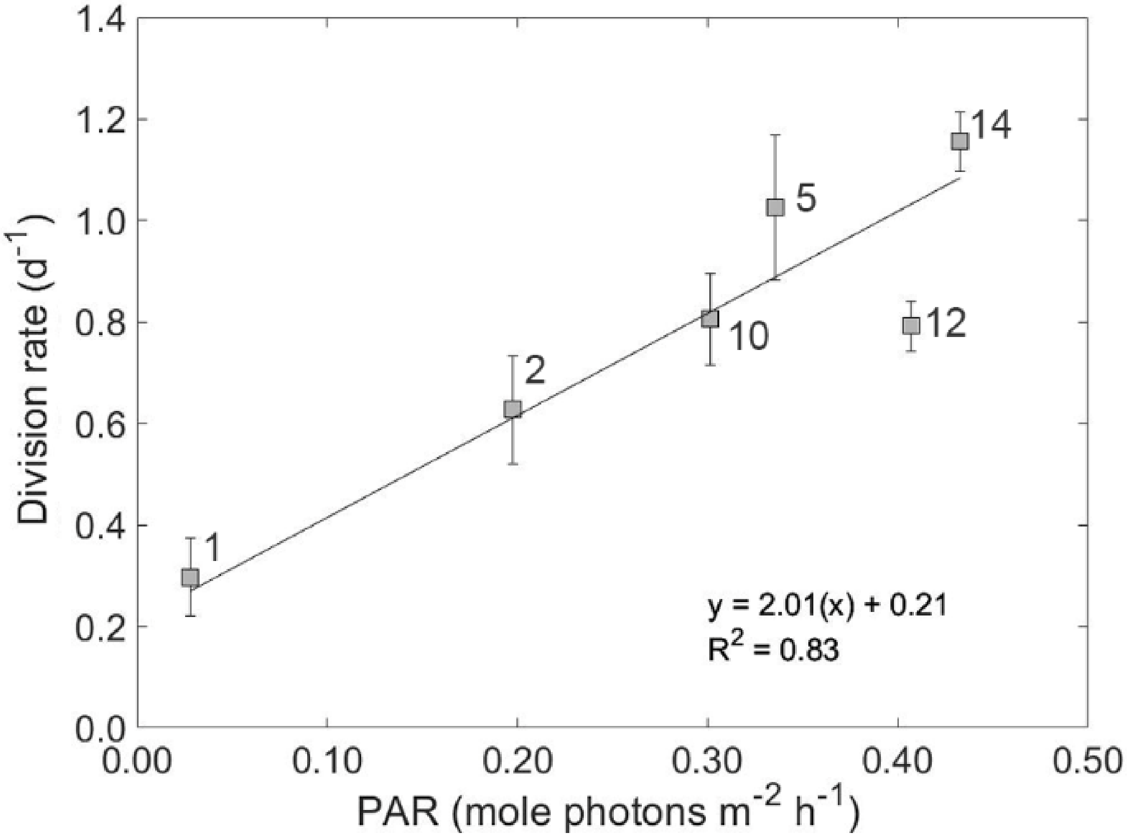

Figure S3. Geometric mean regression of the relationship between average division rate of the phytoplankton community within dilution incubations and PAR levels < 0.5 mole photons m^{-2} h^{-1}. The resulting relationship was used to calculate the growth of the phytoplankton at *in situ* light levels (black squares in Figure 6).
